# Development, Characterization and Electromechanical Actuation Behavior of Ionic Polymer Metal Composite Actuator based on Sulfonated Poly(1,4-phenylene ether-ether-sulfone)/Carbon Nanotubes

**DOI:** 10.1038/s41598-018-28399-6

**Published:** 2018-07-02

**Authors:** Ajahar Khan, Ravi Kant Jain, Priyabrata Banerjee, Bhaskar Ghosh, Inamuddin, Abdullah M. Asiri

**Affiliations:** 1 Department of Surface & Field Robotics Group, CSIR- Central Mechanical Engineering Research Institue (CMERI), Durgapur, 713209 India; 2 Surface Engineering & Tribology Group, CSIR-Central Mechanical Engineering Research Institute, Durgapur, 713209 India; 3 Academy of Scientific and Innovative Research (AcSIR), CSIR-Central Mechanical Engineering Research Institute (CMERI) campus, Durgapur, 713209 India; 4 Chemistry Department, Faculty of Science, King Abdulaziz University, Jeddah, 21589 Saudi Arabia; 5 Centre of Excellence for Advanced Materials Research, King Abdulaziz University, Jeddah, 21589 Saudi Arabia; 6 Advanced Functional Materials Laboratory, Department of Applied Chemistry, Faculty of Engineering and Technology, Aligarh Muslim University, Aligarh, 202002 India

## Abstract

This paper presents the development of new cost-effective hybrid-type sulfonated poly(1,4-phenylene ether-ether-sulfone) (SPEES) and functionalized single-walled carbon nanotubes (SWNT) based actuators produced by the film-casting method followed by chemical reduction of Pt ions as electrodes. The preparation of SPEES was investigated in details and sulfonation of polymer was characterized by ion exchange capacity (IEC), Fourier-transform infrared (FTIR) and degree of sulfonation measurements. SPEES having degree of sulfonation of 126% was blended with SWNT and used to fabricate IPMC actuator. The chemical composition and detailed structure of SPEES-SWNT ionic polymer membranes were confirmed by FTIR, EDX and transmittance electron microscopy (TEM) analysis. Scanning electron microscopy (SEM) micrographs revealed the homogeneously distributed layers of Pt electrodes on the surfaces of IPMC membrane. The electrochemical and electromechanical properties of SPEES-SWNT-Pt-based IPMC actuator shows a better actuation performance than conventional IPMC actuators in terms of higher IEC, Proton conductivity, higher current density, electrochemical impedance spectroscopy (EIS), and large bending deflection. The robust, flexible and mechanically strong membranes prepared by the synergistic combination of SPEES and SWNT may have considerable potential as actuator materials for robotic and biomimetic applications.

## Introduction

Electroactive polymers (EAPs) that responed to electrical stimulation are referred to as smart materials^1,2^. Migration of ions under electrical response within the polymer matrix can be realized by EAPs including ion exchange membranes^3^, solid electrolyte composites containing ionic liquid^4^, conducting polymers^5^ and ionic gels^6^. EAPs based ionic polymer metal composites (IPMCs) with points of interest of simple preparing, adaptability and lightweight^1^, regularly alluded to as ‘artificial muscles’ which have advanced as a promising antecedent in biomedical frameworks and naturally enlivened robots^7–11^. To meet specific requirements electromechanical properties of polymer materials can be fine-tuned during the fabrication of IPMCs actuator. Typically, an IPMC consists of an ion exchange polymer or composite membrane plated by novel metal electrodes infused with a solvent as an inner medium. A voltage difference across the electrodes generates an electric field that initiates the actuation within IPMC membrane. Due to large-scale deflections with modest operating voltage and ability to exhibit bio-mimetic flexibility, contrary to conventional actuator like motors, IPMCs have elevated the prospects of their adoption in robotics applications^12–14^. Despite its slow response time, several intricate schemes and work have been carried out to overcome these routes. In order to establish the use of IPMC as a trustworthy actuator component in soft robotic applications, it is of vital importance to develop an effective IPMC actuator for achieving desired force response and large and fast deflection.

In view of the above discussion, a novel and effective ionic actuator was developed by sandwiching sulfonated poly(1,4-phenylene ether-ether-sulfone)/SWNT (SPEES-SWNT) membrane between platinum metal electrode and its electrochemical characterization and performance at low applied voltage was exploited. The cost-effective poly(1,4-phenylene ether-ether-sulfone) (PEES) is relatively new material to be used in the field of membrane technology. The aromatic and ether linkages present on the polymer backbone provides the necessary strength, molecular rigidity and good processability^15^. However, the hydrophobic nature of PEES restricts the wide-scale utilization of the material in the field of actuators and sensors. Hence, PEES was chemically modified by a sulfonation reaction to increase the much required hydrophilicity so that it can be used for the fabrication of IPMC actuator. Sulfonated poly(1,4-phenylene ether-ether-sulfone) (SPEES) serves as a novel alternative to the commonly used Nafion produced by Dupont, because of its excellent film-forming capability, good chemical, mechanical and thermal stabilities^16,17^. Therefore, SPEES is a potential ionomeric material for the fabrication of IPMC actuators. In order to enhance the electrical properties an effective solution is to incorporate carbon nanotubes (CNTs), which have superior electrical conductivity^18^. CNTs based on a combination of quantum chemical and double-layer electrostatic effects could be excellent electromechanical actuators^19^. The actuation processes of CNTs such as light-driven actuation, pneumatic actuation and electrostatic actuation have also been proposed^20,21^. The chemical functionalization of CNTs which may not only bring this structures into the fold of (macro) molecular chemistry but also afford ultimate new uniqueness has attracted considerable attention^22,23^. On the other hand, functionalization of CNTs can form very stable dispersions, which make them to form homogeneous mixtures with SPEES very easily. It was essential to form homogenous distribution of carbon nanotubes on the fabricated membrane surface for the better actuation performance. Ionic liquids (ILs) are non-volatile wide potential windows, and characterized by their high ionic conductivities, which are likely beneficial for large actuation performance of IPMC actuator in air. IPMCs and conductive polymer based actuators using ILs as electrolytes were reported by several researchers^24–26^. Asaka *et al*.^27,28^ successfully developed bucky-gel (gel-like material) material to fabricate the first-ever dry actuator containing single-walled carbon nanotubes and IL^29^. A combination of large surface area and electrical conductivity of the individual functionalized CNT nanoparticles and excellent electrochemical properties of the composite materials provides good physiochemical properties and actuation performance to the prepared IPMC membranes. The developed SPEES-SWNT-Pt composite based IPMC membrane actuator exhibited higher current density, IEC, mechanical strength, proton conductivity, large and fast bending actuation as compared to previous development^1,30,31^. When operated at low applied voltage, this device inherently was capable of rapid operation with long lifetime.

## Experimental

### Materials

Poly(1,4-phenylene ether-ether-sulfone) pellets (Tg 192 °C), 1-ethyl-3-methylimidazolium tetrachloro aluminate, ≥95% (IL) and N,N-dimethylacetamide, ≥99% (NMA) (Sigma Aldrich, USA), carbon nanotubes, single-walled min. 90%, OD: 1–2 nm, length: 5–30μm (SRL, Pvt. Ltd., India), tetraamineplatinum (II) chloride monohydrate [Pt(NH_3_)_4_Cl_2_·H_2_O (Crystalline)] (AlfaAesar, USA), ammonium hydroxide (25%), hydrochloric acid (67%), sodium nitrate and sodium sulfate (Merk Specialties Pvt Ltd., India), borohydride (NaBH_4_) (Loba Pvt. Ltd. India) were used as received without further purification.

### Reagent solutions

The aqueous solutions of HCl (2 M), NaNO_3_ (1 M), tetraamineplatinum (II) chloride monohydrate (0.04 M), NH_4_OH (5.0%) and NaBH_4_ (5.0%) was prepared using demineralised water (DMW).

### Functionalization of SWNT

**Figure 1:**
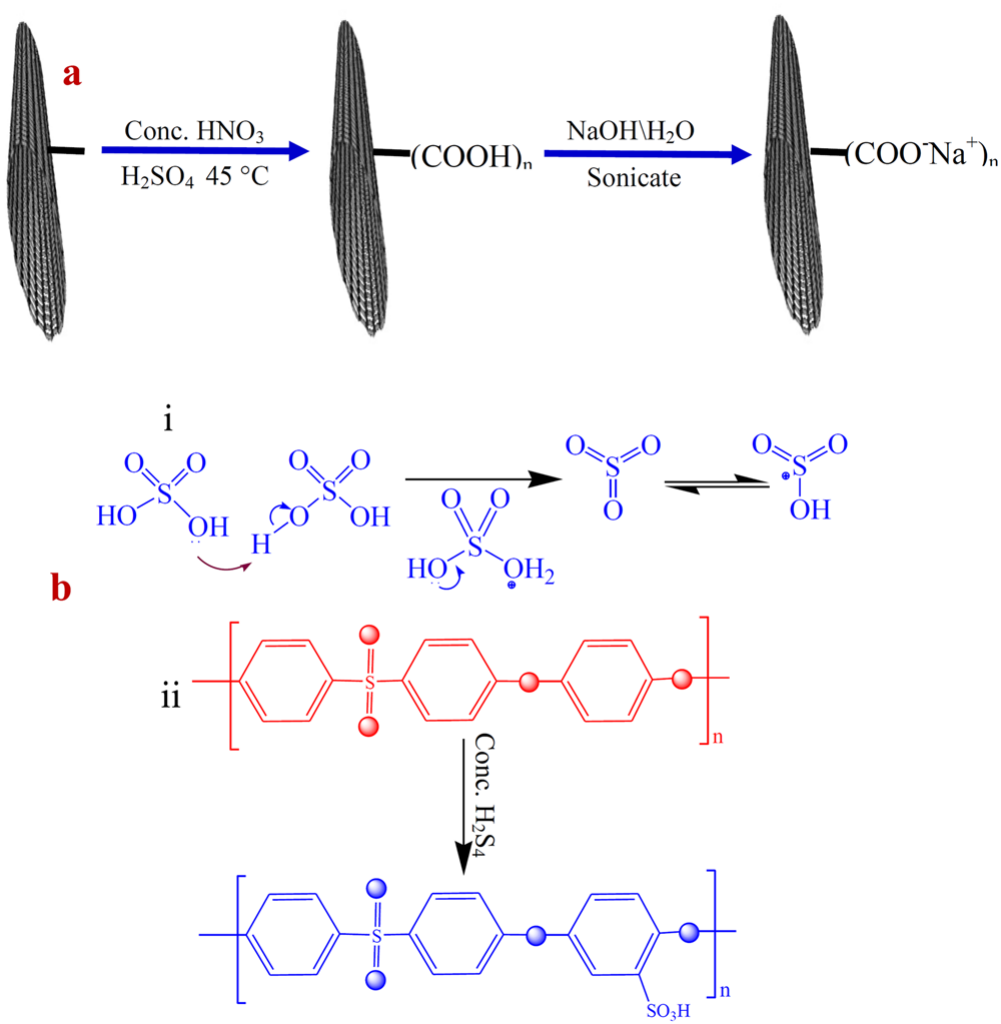
(**a**) Graphical illustration to functionalize carbon nanotubes, (**b**) sulfonation of PEES.

The acid form of commercially available carbon nanotubes was obtained by sonicating in 1:3 (v/v) concentrated nitric acids and sulfuric acid for 4 h at 45 °C. The resultant solid was washed thoroughly several times with DMW until the pH value reaches 6–7. Now, the acid form SWNT-COOH was sonicated in aqueous NaOH (5 mM) for 2 min to convert into the sodium salts (SWNT-COO^−^Na^+^)^32^. After washing, the black solid of SWNT-COO^−^Na^+^ (SWNT) was collected and dried at 50 °C. Finally, functionalized SWNT-COO^−^Na^+^ (250 mg) was added in 10 mL NMA and the mixture was mechanically agitated followed by sonicating for degassetion to make the homogeneous dispersion. Figure 1a shows the graphical concept to functionalize carbon nanotubes.

### Sulfonation of PEES

Sulfonation of base polymer PEES was carried out by a slightly modified method as reported by Unveren *et al*.^33,34^ A 5 g PEES was dissolved completely in excess of concentrated H_2_SO_4_ (125 mL) by strong mechanical agitation (450 rpm) for a time interval of 36 h. Here, concentrated H_2_SO_4_ was used as a solvent as well as sulfonating agent both. After constant stirring up to determined reaction time to obtain white sulfonated PEES (SPEES) strings (Fig. 1b), the polymer solution was gradually precipitated in ice-cold DMW. The decanted sulfonated PEES strings were repeatedly washed several times with DMW until the pH of wash water was approximately 6–7. The SPEES was hydrophilic in nature showed considerable swelling during neutralization process. This swollen sulfonated PEES strings were dried under the thermostated oven for over 24 h at 60 °C and then at room temperature.

### Fabrication of IPMC

To fabricate the SPEES polymer membrane by the conventional solution casting method, 16 wt% of the polymer solution was prepared in NMA at 45 °C under continuous stirring for obtaining a viscous solution. Homogeneous casting blend was prepared by the addition of 200 µL of IL and an appropriate amount of functionalized SWNT dispersion (0.8 g in 25 mL NMA) to the SPEES solution under constant stirring for 24 h. On ensuring complete blend dissolution, the solution was degassed by sonication for 30 min before casting. Then, the composite blend was cast into Petri-dishes and dried in a thermostated oven at 80 °C up-to complete evaporation of the solvent. After that, the dried SPEES/IL/SWNT (SPEES-SWNT) membrane was peeled off and washed with DMW and acetone several times before electroless plating of Pt metal.

To fabricate the IPMC actuator SPEES-SWNT based polymer membrane was sandwiched between conductive metal ions (Pt metal) as an electrode by the electroless plating or chemical reduction method as reported by A. Khan *et al*.^30^. The ion exchange capacity (IEC) and degree of sulfonation% {DS(%)} were determined by titration method as mentioned in the literature^1,35,36^. The IEC and DS(%) were determined by the following equations:

1 $${\rm{IEC}}=\frac{Concentration\,\,of\,NaOH\,\times Volume\,\,of\,\,NaOH}{Weight\,\,of\,\,dry\,\,membrane}.$$

2 $${\rm{DS}}( \% )=\frac{M{W}_{PEES}\times IEC\times 100}{1000-(M{W}_{SPEES}-M{W}_{PEES})\times IEC}.$$

where, MW_PEES_ and MW_SPEES_ are the molecular weights of PEES (324) and SPEES {PEES-SO_3_Na(426)}, respectively.

### Characterizations

The chemical and electromechanical properties, surface morphology and chemical composition of the SPEES-SWNT-Pt composite based IPMCs were studied by a variety of techniques. The water uptake (WU) and proton conductivity (PC) of the developed polymer membranes were measured, as reported by Inamuddin *et al*.^12^. The Fourier transforms infrared spectroscopy (FTIR) of SPEES and SPEES-SWNT membranes were recorded between 500–4000 cm^−1^ using Spectrometer (Perkin Elmer Spectrometer). To observe the surface morphology and the cross-sectional view of SPEES-SWNT-Pt IPMC scanning electron microscope (SEM Jeol, JSM-6510LV, Japan) was used. Energy dispersive X-ray (Oxford instruments INCA, X.act, S.No. 56756, UK) analysis was used to determine the elemental composition. Transmission electron microscopy (TEM Jeol, JEM-2100, Japan) was used to observe detailed structure of SPEES-SWNT-Pt-based IPMC membrane. UV-visible absorption spectroscopy of PEES, SPEES and SPEES-SWNT was employed using Perkin Elmer spectrophotometer, Lambda 25. The mechanical stability was determined by a universal testing machine (Model: H50 KS, Shimadzu Corp.), with the 25 mm gauge length between the grips under the testing speed of 5 mm min^−1^. To investigate the electrical property of the proposed IPMC membrane electrochemical impedance spectroscopy (EIS), cyclic voltammetry (CV), linear sweep voltammetry (LSV) at triangle voltage input of ±2 V with a step of 50 mV s^−1^ were performed with Autolab 302 N modular potentiostat/galvanostat. Before electromechanical characterizations, the SPEES-SWNT-Pt IPMC membranes (1 × 3 cm^2^) were immersed in aqueous solution of 0.2 M NaOH at room temperature for 6 h so that the counter cations were exchanged to Na^+^. The successive steps of bending response at 0–4.5 V DC and overtime at 4.5 V were analyzed using a laser displacement sensor (model: OADM 20S4460/S14F; Baumer Electronic, Germany). To find out the repeatability and to check the performance of the proposed IPMC multiple experiments were conducted such as bending actuation, deflection hysteresis, normal distribution and load characterization.

## Results and Discussion

**Figure 2:**
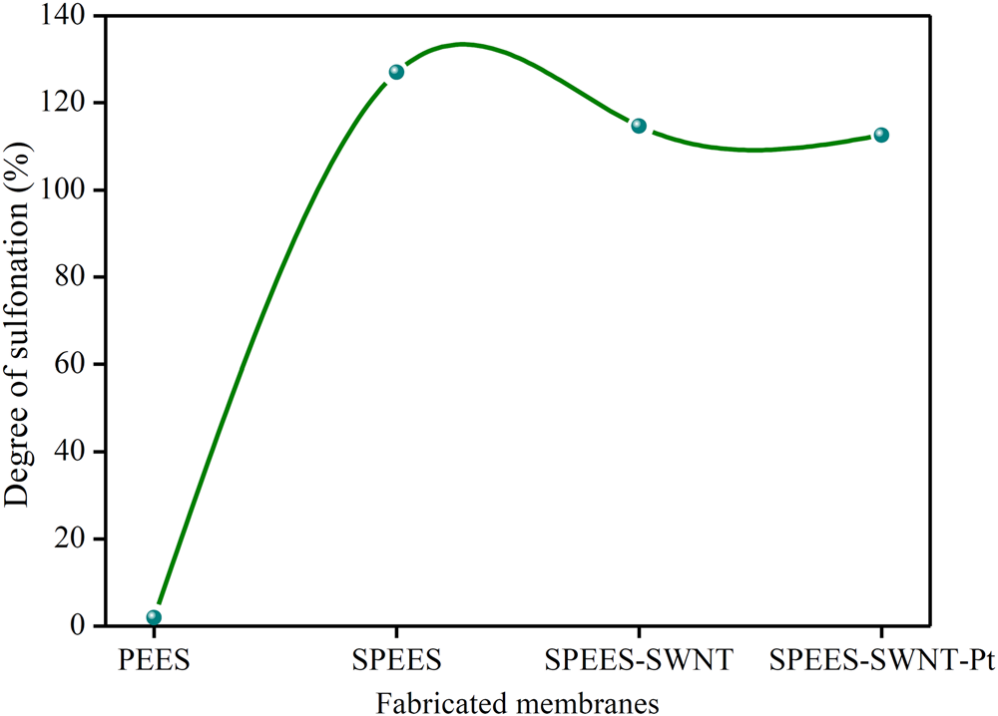
DS(%) of pure SPEES, SPEES-SWNT and SPEES-SWNT-Pt composite polymer membranes.

To obtain ionic conductivity in thermo-stable hydrophobic PEES polymer, sulfonic acid groups (-SO_3_H) were introduced to the aromatic, non-fluorinated backbone by a sulfonation reaction. The electrophile is generated (Fig. 1(b-i)) and an electrophilic substitution reaction takes place at one of the four positions of the aromatic ring between ether bridges as shown in the SPEES repeat unit in Fig. 1(b-ii). The electron-attracting nature of the neighbouring sulfonyl group lowers the electron density of other two aromatic rings in the repeat units. The electron-rich benzene ring then reacts with the electrophile to give the sulfonated product. The DS(%) of pure SPEES, SPEES-SWNT and SPEES-SWNT-Pt IPMC was found to be 126, 114 and 112, respectively, (Fig. 2) which was high enough to enable sufficiently high proton conduction. Noticeable changes in the DS(%) of different composite membranes were known to occur by the introduction of the SWNT and Pt metal in the polymer. It was observed that with an increase in DS(%), the SPEES polymer exhibited excessive swelling as well as partial dissolution in water. To increase the mechanical stability functionalized SWNT was added to the SPEES solution during fabrication of ionic polymer composite as discussed in experimental section. To check the stability, the SPEES-SWNT ionomeric membrane was immersed in hot water (80 °C) for 24 h and it was found that membrane was completely stable for further characterization. This observation indicated that SPEES-SWNT with higher DS (%) was suitable for membrane-based IPMC actuators and its applications.

### WU, IEC and PC

**Figure 3:**
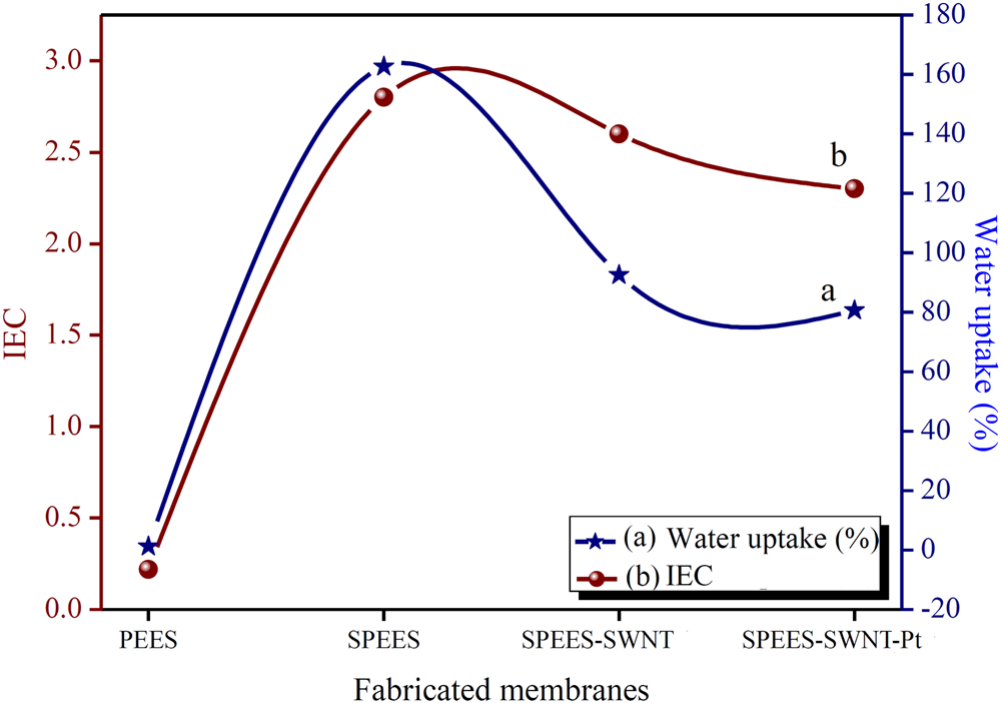
(**a**) WU and (**b**) IEC of PEES, SPEES, SPEES-SWNT and SPEES-SWNT-Pt composite polymer membranes.

Water uptake % (WU) of polymer membranes is a critical property having a good effect to improve the proton conductivity of the ionic membrane^37,38^ and dielectric constant of the IPMC membrane^39^. The proton conductivity (PC) increases with the increase in the WU of polymer membranes at the same time mechanical stabilities of the membrane decline. An elevated WU is vital for actuation of an IPMC membrane, because of the movement of the hydrated cations along with the water molecules, when low electric potential are applied to the IPMC. Figure 3(a) shows that the maximum WU for SPEES, SPEES-SWNT and SPEES-SWNT-Pt polymeric membranes were found to be 162, 92 and 80%, respectively, after immersion in DMW for 24 h. It can be clearly seen from the Fig. 3(a). The WU of the pure SPEES is much more than blended SPEES-SWNT and SPEES-SWNT-Pt ionomeric membranes due to the high IEC (Fig. 3(a)). Compared to pure SPEES membrane, the SPEES-SWNT and SPEES-SWNT-Pt membranes exhibited lower WU. The addition of SWNT decreases the porosity and number of acidic sites and hence the capacity of the membrane to retain the water decreases with the increase in the mechanical stability. Notably, WU of final SPEES-SWNT-Pt IPMC membrane was much higher than those of Nafion based IPMCs^1^. It was very important for improving the performance of IPMC actuator.

The higher IEC of the polymer composite materials means a larger amount of water molecules moves toward the negative electrode by the action of hydrated cations, electro-osmosis and electrophoresis cause greater volume expansion towards cathode side resulting into consequent larger bending deformation^40,41^. The high IEC enables that Pt particles can more easily be embedded deep within the porous surface by the chemical reduction plating method and reduce the resistance, increase the electric current^42^, and the capacitance of an IPMC membrane actuator^42–44^. It can be seen from the Fig. 3(b) that the IEC value of SPEES, SPEES-SWNT and SPEES-SWNT-Pt found to be 2.8, 2.6 and 2.5 meq/g of dry membrane respectively. SPEES-SWNT-Pt-based IPMC membrane has higher IEC than Nafion based IPMC membrane^1^. This was because SPEES-SWNT-Pt membrane actuator has a larger number of sulfonic acid groups. The data obtained from the analysis of IEC and WU of the fabricated SPEES-SWNT-Pt polymer based IPMC actuator (Fig. 3) revealed that the proposed actuator have higher water and ionic content compared to Nafion based IPMC actuator^1^. Higher IEC and WU of SPEES-SWNT-Pt polymer based IPMC actuator confirm the better performance relative to other conventional polymer-based IPMC actuator. The maximum PC of SPEES-SWNT-Pt-based IPMC was found to be 2.321 × 10^−2^ S cm^−1^ (Supplementary Table 1). The high PC of the SPEES-SWNT-Pt membrane enables the better performance because cations move quickly towards the cathode, to create an imbalance pressure inside the membrane. Thus, the rate of actuation of the IPMC membrane is increased with the high PC, which leads to more hydrated cations move quickly toward the cathode side, showing a large displacement and fast actuation^1^. The electric current of an IPMC increases with the increase of proton conductivity of ion exchange polymer membrane. It has been reported that high capacitances and electric current increase the actuation under applied AC and DC voltages^44–46^.

### FTIR study

**Figure 4:**
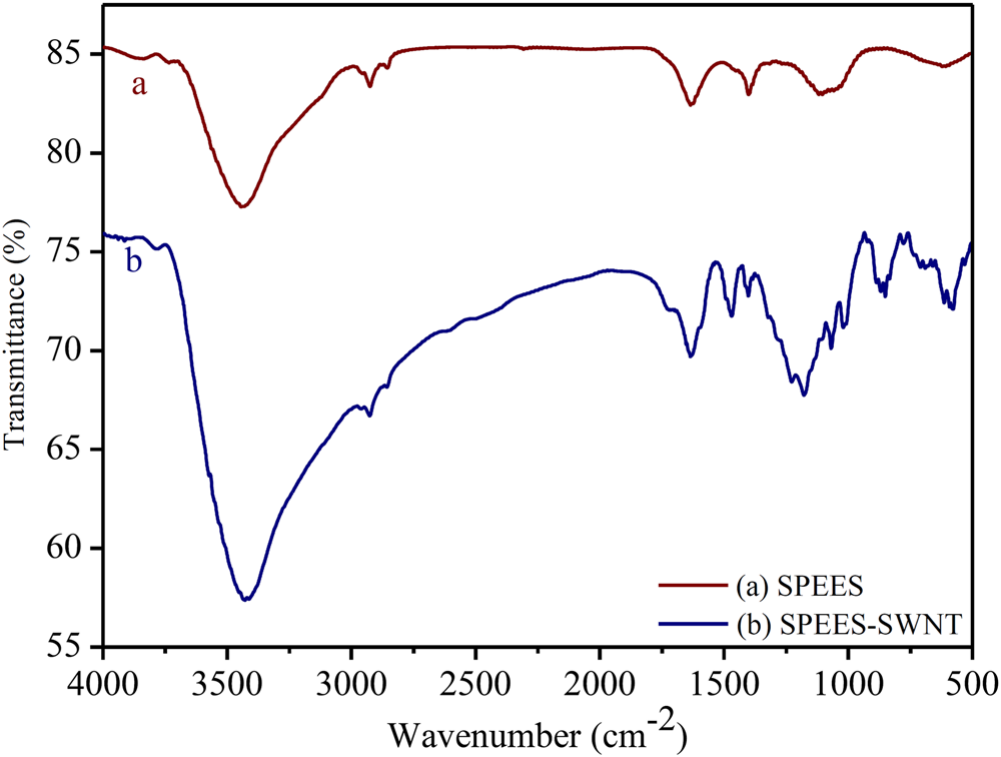
FTIR spectra of (**a**) SPEES and (**b**) SPEES-SWNT.

FTIR spectroscopy analysis was conducted on the SPEES and SPEES-SWNT polymer membranes to confirm the successful introduction of the sulfonic acid group to the PEES and functionalization of carbon nanotubes. The absorption band at 3434 cm^−1^ is due to the O-H stretch of -SO_3_H and absorbed moisture (Fig. 4(a)). The characteristic absorption peaks of -SO_3_H appear at 1089 and 1034 cm^−1^ indicating the existence of O=S=O and S=O stretches respectively. As shown in Fig. 4(b), the absorption bands at 1210 and 1580 cm^−1^ could be assigned to the C-O stretch of the acid and C=C stretch of SWNT backbones, respectively^47^. The absorption peak at 1720 cm^−1^ confirms the existence of the carboxylic acid groups (Fig. 4(b))^47,48^. The absorption bands at 1574 and 1378 cm^−1^ suggest theformation of SWNT-COO^−^Na^+^ (Fig. 1(a))^49^. The characteristic absorption bands of carboxylate salt may also be overlapped with the C=C stretch of the SWNT backbones.

### Tensile Strength

**Figure 5:**
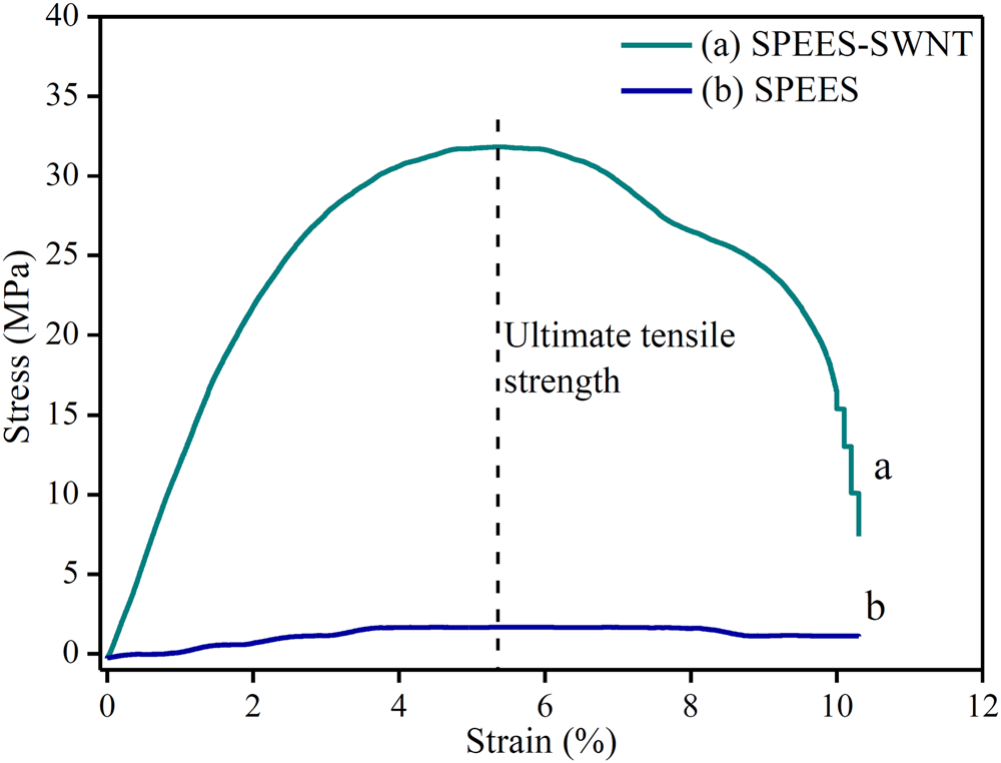
Stress-strain curve of pure SPEES and SPEES-SWNT membranes.

The mechanical properties of the fabricated polymer composite membrane such as Young’s modulus and tensile strength play a significant role on the actuation performance of IPMC actuator. Both the SPEES and SPEES-SWNT-Pt membranes of sizes 20 mm width and 0.15 mm thickness were fixed into UTM machine with fixed gauge length of 25 mm. As the membranes were elongated with loads the displacement of two end points was continuously recorded to attain the mechanical strength. Figure 5(a,b) shows the stress-strain curve of the SPEES and SPEES-SWNT composite membranes and their mechanical properties are shown in Table 1. The Young’s modulus and ultimate tensile strength of SPEES and SPEES-SWNT composite membranes have been calculated by the stress-strain curve as given in Fig. 5. To find out the value of Young’s modulus, the slope of stress vs. strain curve was used. The ten slope values at different point of stress-strain curve were taken from starting point to maximum stress value. Subsequently, the average of these ten slope values was used to calculate the Young’s Modulus of fabricated ionomeric membranes. The ultimate tensile strength was calculated at maximum stress point. As shown in the Table 1, Young’s modulus and ultimate tensile strength of pure SPEES membrane was 88.77 MPa and 1.8 MPa, respectively, whereas 932.62 MPa and 31.8 MPa, respectively, for SPEES-SWNT composite membrane. The results demonstrate that the composite blend of SPEES-SWNT membrane provide high molecular rigidity and mechanical strength which leads to higher tensile moduli relative to the SPEES membrane (Fig. 5). The SPEES-SWNT shows the improved mechanical properties than the pristine SPEES polymer membrane because SWNT have excellent mechanical strength as well as superior electrical properties.

**Table 1:** Mechanical properties of SPEES and SPEES-SWNT ionomeric polymer membranes.

Membranes	Young’s modulus (MPa)	Ultimate tensile strength (MPa)	Elongation at break (%)
SPEES-SWNT	932.6	31.8	10.1
SPEES	88.77	1.8	10.3

### SEM, EDX, TEM studies

**Figure 6:**
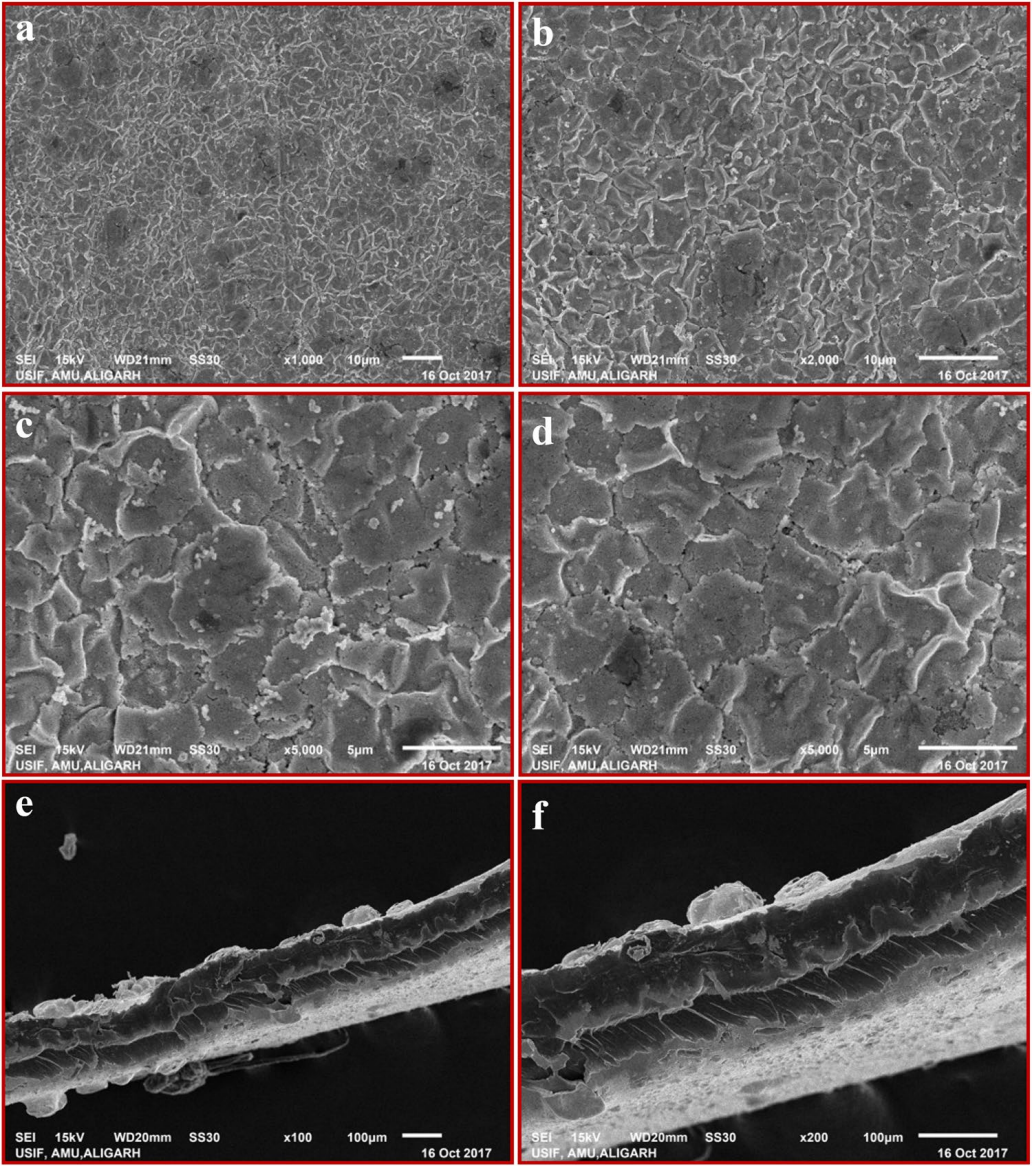
SEM images of SPEES-SWNT-Pt IPMC membrane.

Figure 6(a–f) show the surface and cross-sectional morphologies of the SPEES-SWNT-Pt composite polymer based IPMC membranes. As shown in Fig. 6(a,b) at the surface of SPEES-SWNT membrane the Pt particles homogeneously distributed and covered the whole interface boundaries of IPMC membrane. Figure 6(c,d), clearly shows that Pt particles as an electrode are densely deposited into the surface of the SPEES-SWNT IPMC actuator. The Pt diffusion layers can enhance the interfacial adhesion between the electrode and polymer membrane in this IPMC actuator. Some microcracks are clearly shown on the SPEES-SWNT-Pt IPMC membrane surface as a result of the cracking of the Pt electrode layers during the sample drying before SEM analysis. The cross-sectional micrographs show the two layers of Pt electrodes deposited on both the surfaces of SPEES-SWNT composite membrane (Fig. 6 (e,f)).

**Figure 7:**
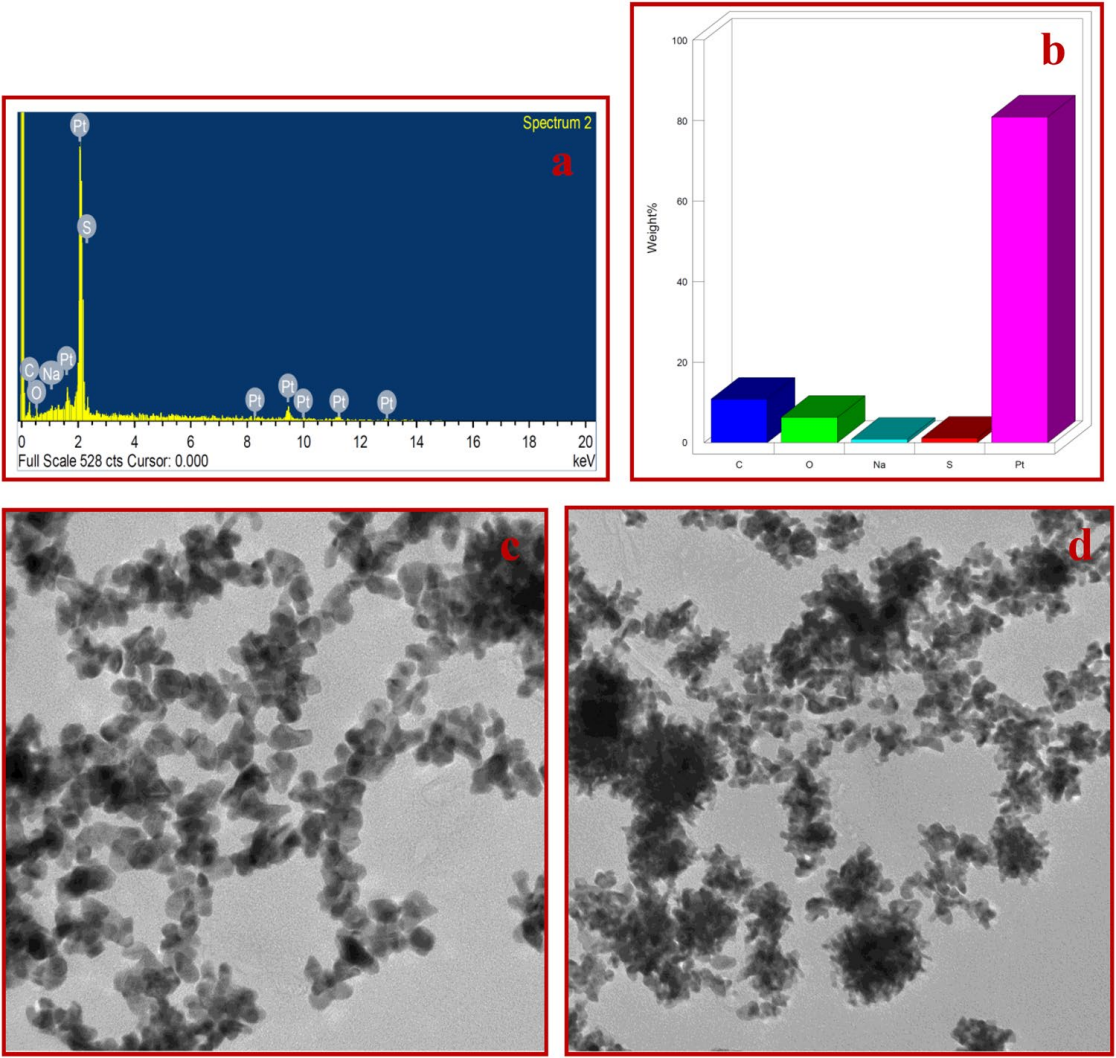
EDX graphs (**a**,**b**) and TEM micrograph (**c**,**d**) of SPEES-SWNT-Pt IPMC membrane actuator.

EDX was analyzed to confirm the composition of developed SPEES-SWNT-Pt membrane based IPMC actuator. Figure 7(a,b) show the results of the EDX analysis. The EDX spectrum of the SPEES-SWNT-Pt membrane surface shows the characteristic peaks of elements (Fig. 7(a)), while Fig. 7(b) show the composition of carbon, oxygen, sulphur, chlorine and platinum at the surface of the fabricated the IPMC actuator. The characteristic peaks of Na element (Fig. 7(a)) confirm the functionalization of SWNT into SWNT-COO^−^Na^+^ (Fig. 1(a)). The large amount of the Pt on the surface confirm the excellent and uniform coating of metal electrode on the surface of SPEES-SWNT-Pt-based IPMC membrane, which suggest the better performance of proposed actuator. TEM micrographs of the SPEES-SWNT-Pt-based IPMC membranes are shown in Fig. 7(c,d). In Fig. 7(c), the black spots show SPEES on grey background of alkyl-modified SWNT nanotubes. Figure 7(d) clearly shows that the Pt as electrode homogeneously distributed and covered grey background of SPEES and SWNT matrix of SPEES-SWNT-Pt IPMC membrane. The TEM image confirms the existence and homogeneous composite blend of the SPEES, SWNT and Pt electrode in the fabricated IPMC membrane.

### Porosity

**Figure 8:**
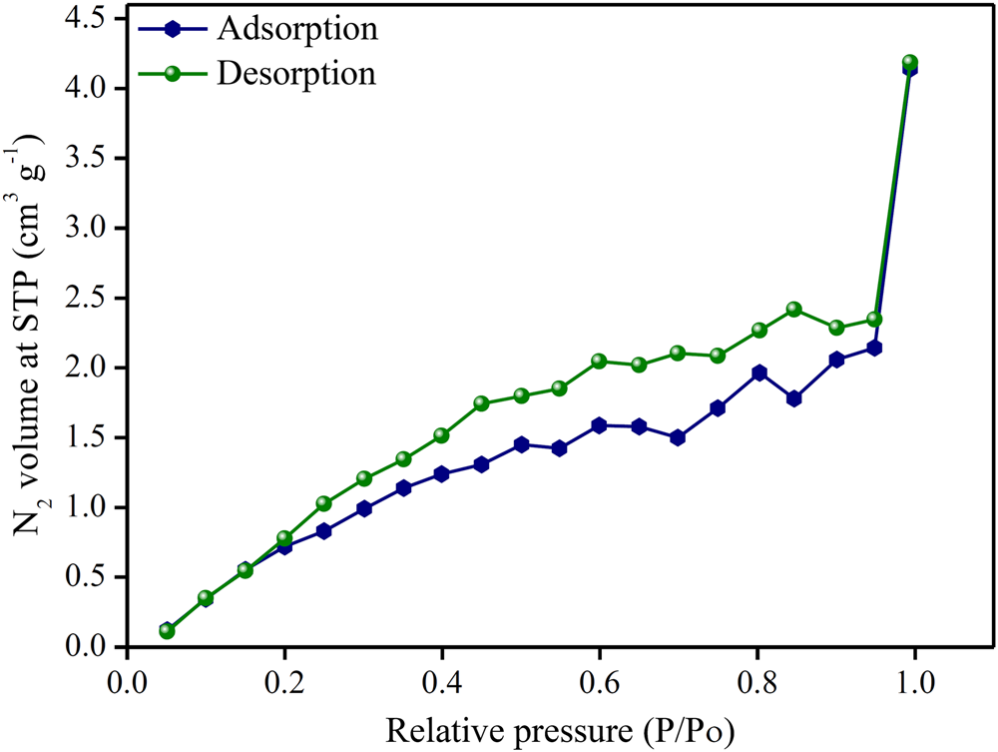
BET analysis of SPEES polymer membrane.

The BET analysis was used to study the porous nature of SPEES membrane as shown in Fig. 8. The ionic polymer SPEES membrane exhibited the typical N_2_ adsorption-desorption curve for porous materials. The total pore volume and surface area of the SPEES ionic polymer membrane were found to be 6.477 × 10^−3^ cm^3^g^−1^ and 16.970 m^2^g^−1^, respectively. In addition, the BET results confirm the porous structure of the SPEES membrane, which facilitates ion transport within the ionic polymer membrane, thus leading to high-performance IPMC actuator.

### UV-visible studies

**Figure 9:**
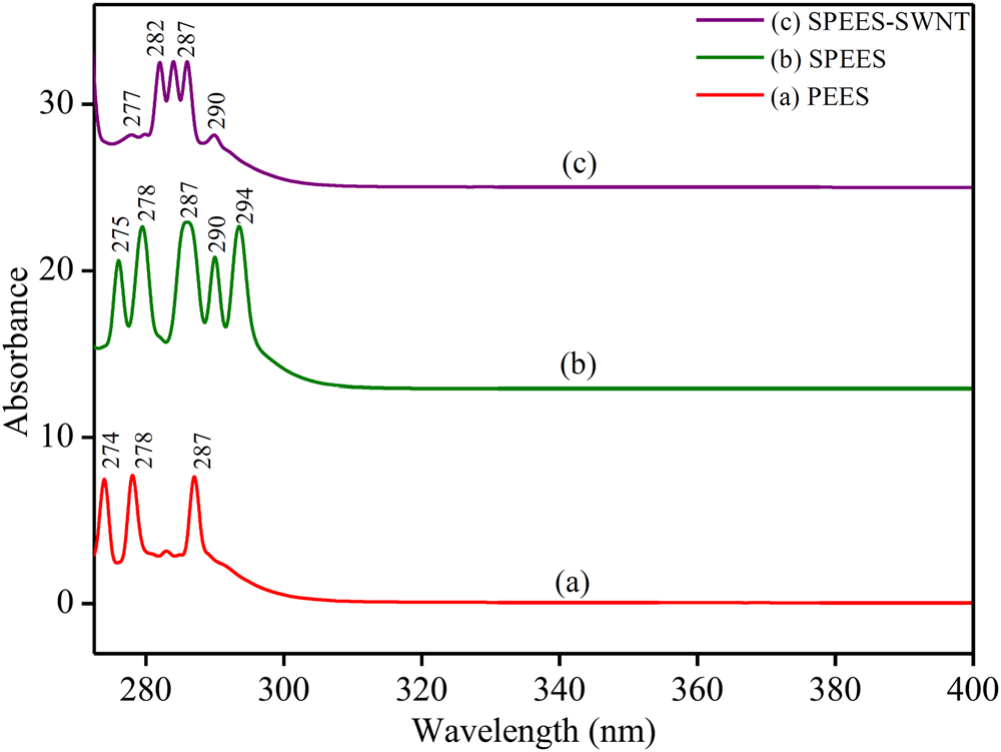
UV-visible pattern of (**a**) PEES, (**b**) SPEES and (c) SPEES-SWNT membranes.

As shown in Fig. 9(a–c), the UV-visible absorbance patterns of PEES, SPEES and SPEES-SWNT determine the chemical interaction between SPEES and SWNT composite in the developed IPMC membrane. Figure 9(a) shows the absorbance peaks at 274, 278 and 287 nm may be attributed due to the characteristic absorbance peaks of PEES. Figure 9(b) shows the additional absorbance peaks at 290 and 294 nm which was appeared after sulfonating the PEES polymer, which was used as base ion exchange polymer for the fabrication of IPMC actuator. Figure 9(c) shows the absorbance pattern of SPEES-SWNT-Pt-based IPMC in which an additional absorbance peak at 282 nm with the diminishing of other peaks appear in Fig. 9(a,b). This confirms the chemical interaction between SPEES and SWNT in IPMC membrane. The additional absorbance peaks with the disappearing/minimizing or overlapping of the other peaks after addition of SWNT and Pt give the evidence of chemical interaction between SPEES, SWNT and Pt-based composite used for the fabrication of IPMC actuator.

### Thermal analysis

**Figure 10:**
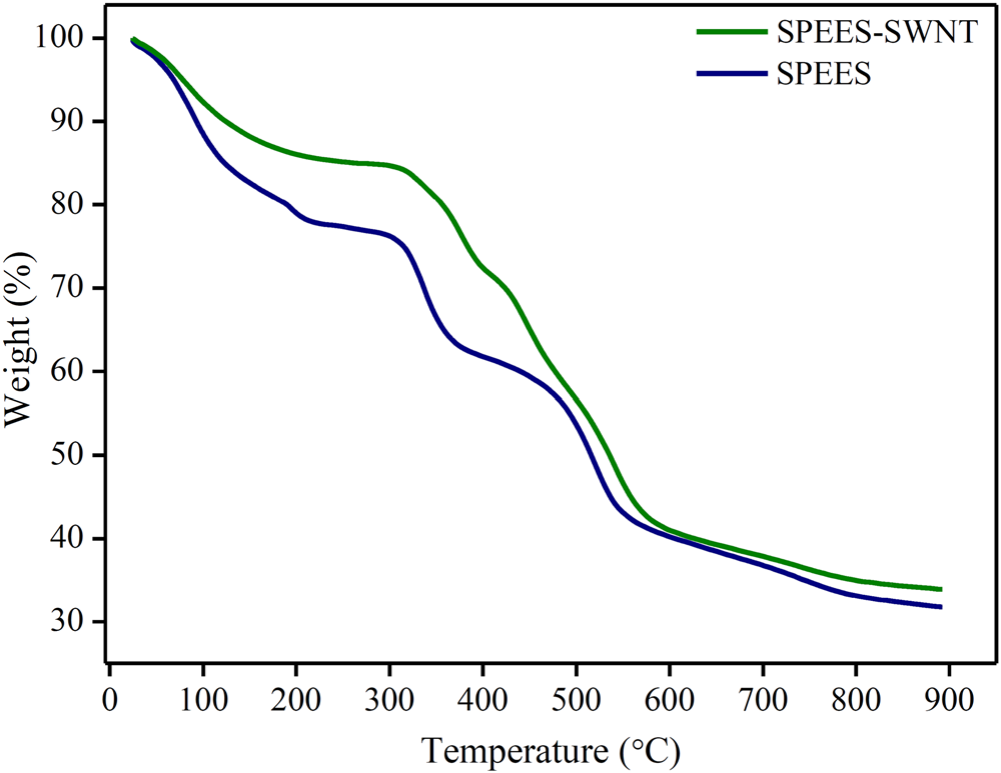
Thermal gravimetric analysis of SPEES and SPEES-SWNT ionic polymer membranes.

Figure 10 shows the thermal studies of SPEES and SPEES-SWNT ionic polymer membranes examined by TGA analysis. Both the ionic polymer membranes have three stages of weight loss. The initial weight loss in SPEES and SPEES-SWNT membranes occurs at the temperature of 50–200 °C which was due to the removal of water molecules absorbed or bonded to the sulfonic acid groups in the ion exchange polymer membranes. The second stage located at 300–450 °C resulted from the sulfonic acid groups in the SPEES and SPEES-SWNT. The third weight loss around 500 °C onwards assigned to the degradation of polymer backbones. The thermogravimetric analysis confirms that the SPEES-SWNT composite polymer membrane exhibits higher thermal stability than pristine SPEES (Fig. 10). This suggested the better performance of the SPEES-SWNT ionic polymer membrane even at a higher temperature.

### Electrochemical characterization

**Figure 11:**
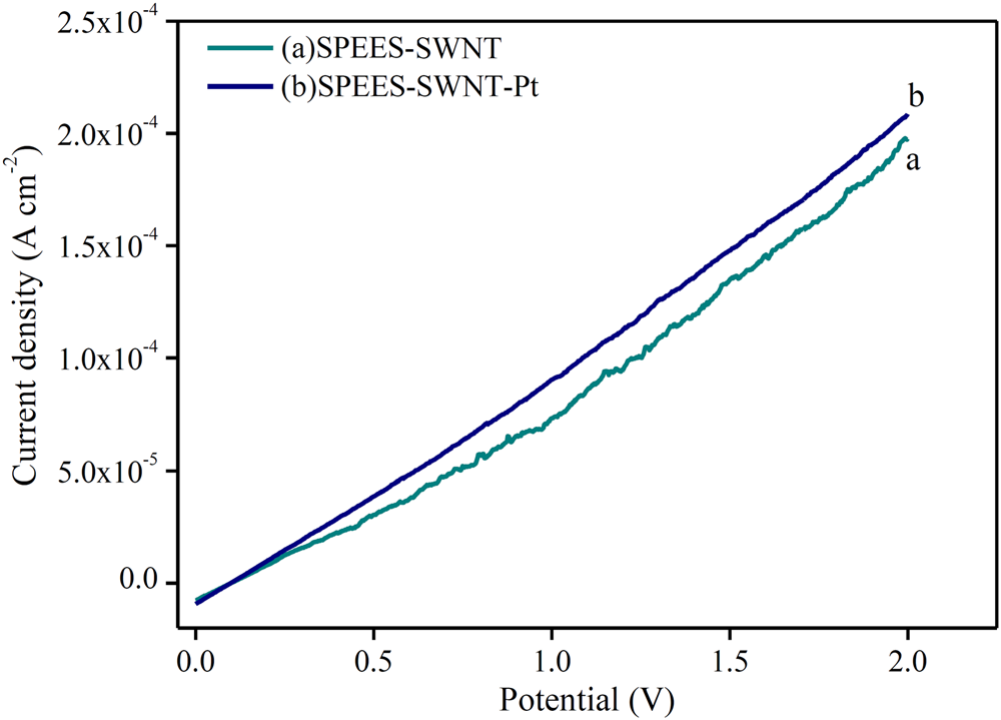
LSV analysis of SPEES-SWNT and SPEES-SWNT-Pt membrane actuators.

**Figure 12:**
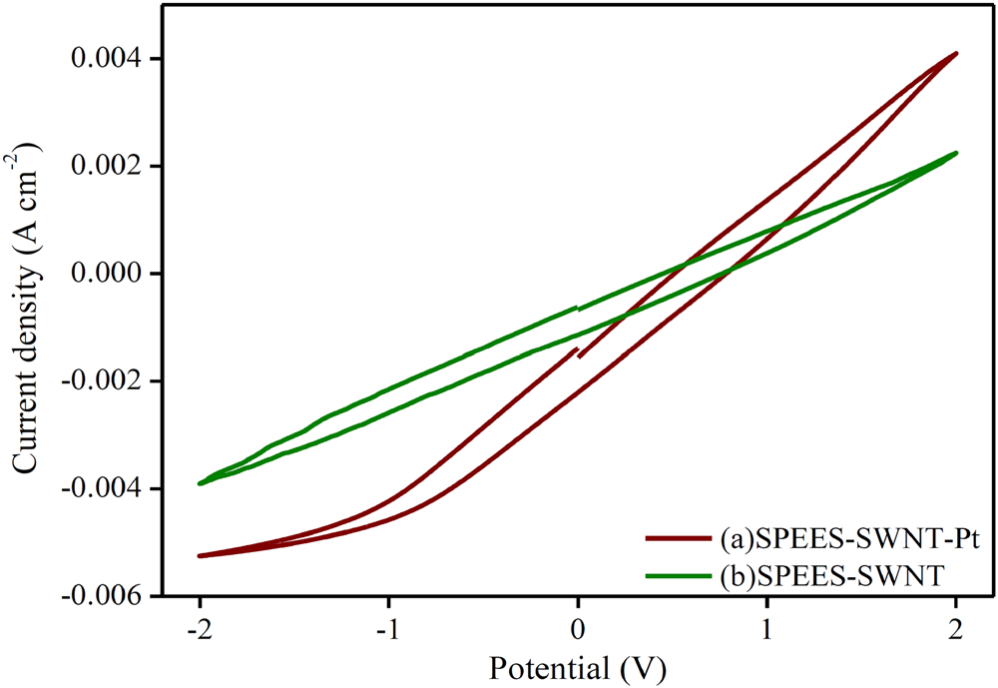
CV curves of SPEES-SWNT and SPEES-SWNT-Pt membrane actuators.

The electrochemical studies of the SPEES-SWNT and SPEES-SWNT-Pt-based IPMC membranes were demonstrated by current-voltage hysteresis curve CV and LSV at triangle voltage ±2 V under scan rate of 50 mVs^−1^ in a three-electrode system. The LSV curve shown in Fig. 11 reveals that the SPEES-SWNT-Pt IPMC shows the higher value of current density than SPEES-SWNT, which proves that the electrical current directly depends on the types of electrode layer deposited on the surface of IPMC membrane under the same applied voltage. Hence, the rate of ionic transfer was higher in SPEES-SWNT-Pt IPMC actuator as compared to the SPEES-SWNT composite membrane due to the presence of metal electrode. Figure 11(b) shows that the LSV curve of SPEES-SWNT with different sequential small peaks have an irregular shape. This indicates that ionic diffusion in the composite membrane was chaotic which may be attributed due to rough and porous surface (Fig. 6(c,d))^50,51^. The I-V hysteresis curves of SPEES-SWNT and SPEES-SWNT-Pt membranes are appearing similar in shapes but different in the magnitude of the current density (Fig. 12). The symmetric shape of the CV curves can be assigned to excellent charge distribution in the whole surface region of the developed IPMC (Fig. 12). The observed current density of the fabricated SPEES-SWNT-Pt-based IPMC membrane was remarkably higher than several other reported so far IPMC,^1,12,30^. The higher current density in the fabricated IPMC may be due to the addition of electrically conductive alkyl-modified SWNT, which increase the charge transfer due to increase in surface area for chemical reaction. The benefits of the higher IEC, PC, increased conductivity and uniform electroding of Pt was indicated by the more capacitor like the shape of the I-E curve of SPEES-SWNT-Pt IPMC membrane. The energy storage ability of the IPMC is reflected by the higher current density, which is responsible for the better actuation performance by larger deformation of polymer membrane under applied voltage.

### Electrochemical Impedance Spectroscopy (EIS)

**Figure 13:**
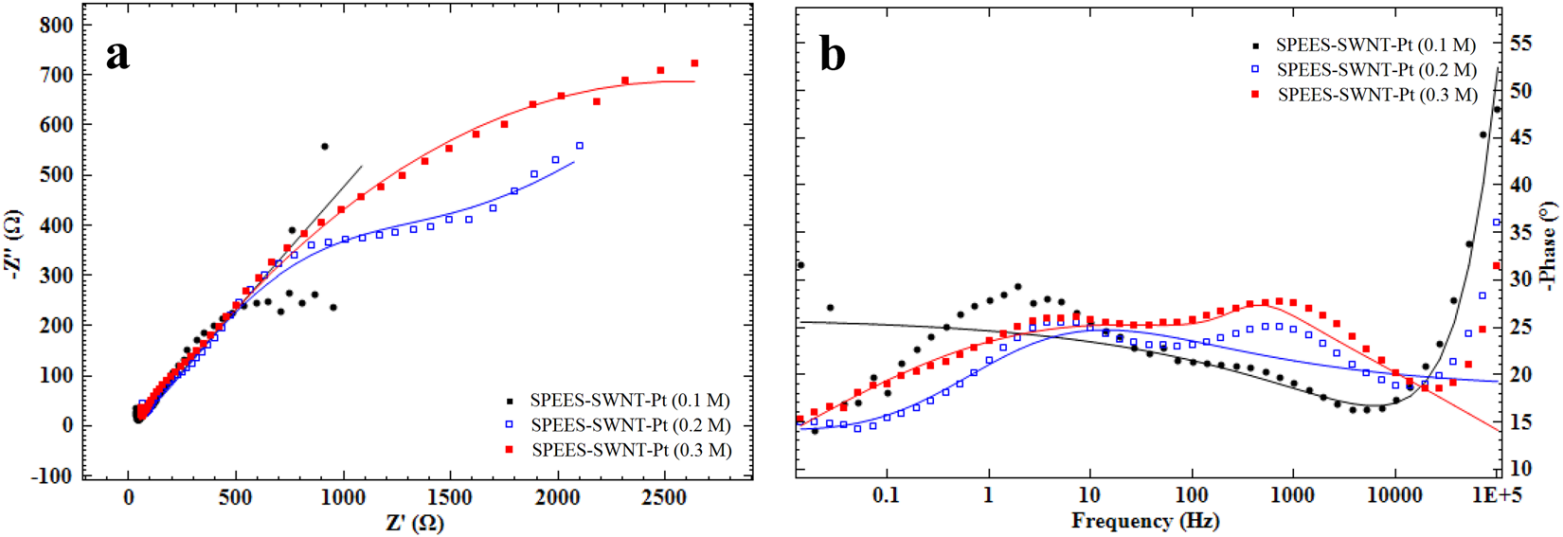
(**a**) Nyquist plot and (**b**) Bode phase plot for SPEES-SWNT-Pt.

EIS is the powerful tool to investigate the electrochemical properties of an IPMC actuator. Figure 13(a,b) represent the Nyquist and Bode phase plots of SPEES-SWNT-Pt membrane actuator in 0.1,0.2 and 0.3 M sodium sulfate solution. It was observed that in all the three concentrations of salt solutions, the Nyquist plots show similar behavior and have semicircle shapes in the high frequency region (Fig. 13(a)). The smallest semicircle obtained at the far left of the x-axis due to Pt used for electroding, implying the lowest polarization resistance of SPEES-SWNT-Pt-based IPMC membrane actuator. But with the increase in the concentration of salt solution from 0.1 to 0.3 M, Nyquist plot (shown in Fig. 13(a)) for SPEES-SWNT-Pt membrane actuator become nearly linear in the low frequency region. This suggests that at higher concentration of salt solution the SPEES-SWNT-Pt IPMC membrane possess the highest charge storage capacity with the highest charge transfer resistance. The low value of ionic resistance of the SPEES-SWNT-Pt IPMC membrane at the high frequency intercepts (X-axis) showed due to its higher IEC and WUP (Table 2). EIS results also reveal that electrolyte concentration has an enhancing impact on the capacitive characteristic of the proposed actuator. The frequency response of SPEES-SWNT-Pt membrane as a function of the electrode structure was analyzed by the Bode phase plots (Fig. 13(b)). All the Bode phase plots exhibit two peaks and show approximately similar behavior, which were assigned as two time constants and indicating the diffusion-limited mass transport of the SPEES-SWNT-Pt membrane actuator at low frequency regions^52^. The EIS data was than fit with the most satisfactory equivalent circuit model to estimate the electrochemical parameters are depicted in Supplementary Fig. 1. The chosen equivalent circuit for SPEES based IPMC membrane composed of solution resistance (R_s_) and constant phase elements (CPE) and charge transfer resistance (R_ct_). CPE a non-ideal capacitor substituted for double layer capacitance (C_dl_) and its impedance can be calculated according to equation (3)^53,54^.

3 $${{\rm{Z}}}_{{\rm{CPE}}}=\frac{1}{{{\rm{Y}}}_{{\rm{o}}}{(j{\rm{\omega }})}^{{\rm{n}}}}$$

where Y_o_ is the amplitude comparable to the CPE and n is the CPE exponent (shows the roughness of the electrode with values varies from 0 to 1). An ideal capacitor shows n = 1, while n = 0 shows resistor, n = −1, then CPE resembles an inductor and for n = 0.5 the Warburg diffusion behavior^54,55^, j is the square root of −1 (imaginary number) and ω represent angular frequency in Radius^−1^. The parameters of the equivalent circuit for SPEES-SWNT-Pt IPMC membrane are given in Table 2. The high value of n for the proposed IPMC actuator indicates the finest electrode structure with surface porosity. For the SPEES-SWNT-Pt base actuator the true capacitance i.e. the double layer capacitance (C_dl_) was calculated from the equation (4):

4 $${C}_{dl}={Y}_{0}{({\omega }_{max})}^{{\rm{n}}-1}$$

where ω_max_ = 2π*f*_*max*_ (*f*_*max*_ denotes maximum frequency at which the imaginary component of the impedance has a maximum value). The high value of C_dl_ for SPEES-SWNT-Pt IPMC membrane assigned due to the high ionic and water content and surface porosity of the electrode region (Table 2). Moreover, the actuator showed the high value of R_ct_ which may be due to the blending of SWNT with the SPEES ion exchange polymer membrane.

**Table 2:** EIS parameters for SPEES-SWNT-Pt-based membrane actuator in varying concentrations of sodium salt solutions.

Concentration (M)	R_s_ (Ω cm^2^)	R_ct_ (Ω cm^2^)	CPE		*F*_*max*_	C_dl_ (F)
			Y (F)	n		
0.1	31.759	1354.5	0.001224	0.99046	0.193	0.001222
0.2	40.704	2655.5	0.000365	0.98939	0.099	0.000367
0.3	9.1179	4924.4	0.001639	0.98832	0.072	0.001654

### Electromechanical characterization

**Figure 14:**
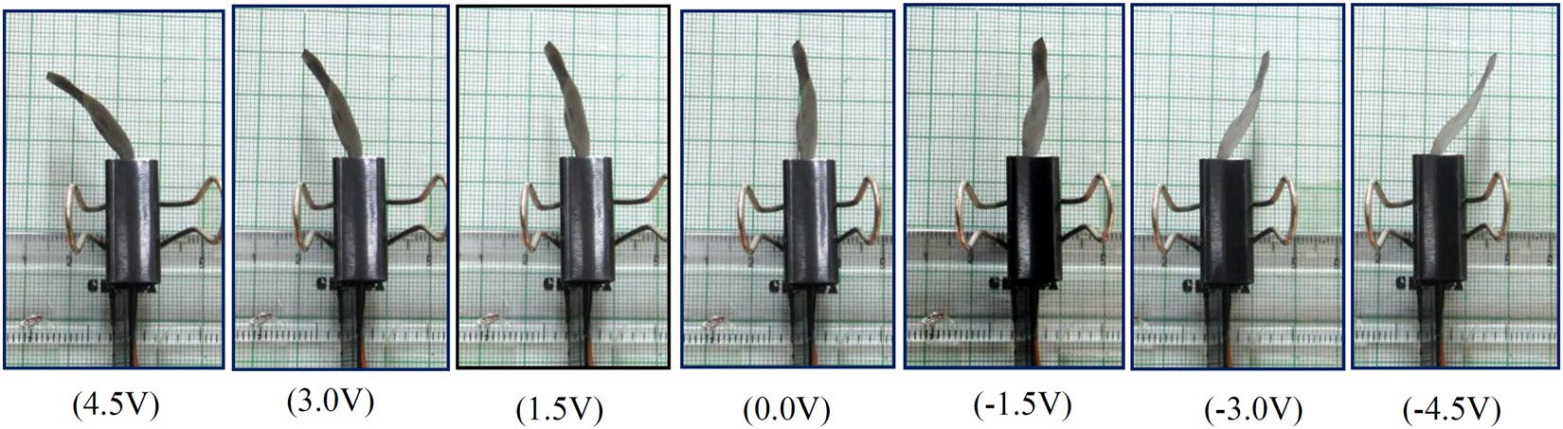
Deflection images of SPEES-SWNT-Pt-based IPMC membrane.

**Figure 15:**
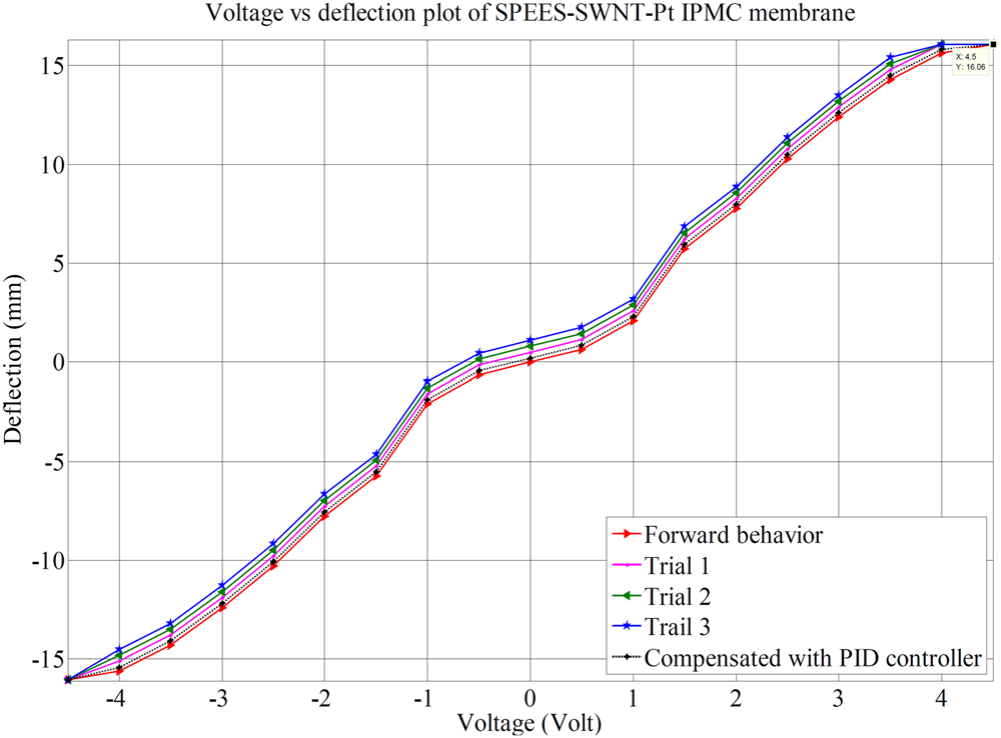
Deflection behaviour of SPEES-SWNT-Pt-based IPMC for hysteresis analysis.

An actual testing setup was developed to investigate the behavior of SPEES-SWNT-Pt-based IPMC membrane (Supplementary Fig. 2). The fabricated IPMC was clamped and connected to digital power supply and NI-PXI System. This sent the controlled voltage (±4.5 V) to IPMC membrane. For controlling this voltage, a VI was designed in Lab view software where proportional integral derivative (PID) control system feature enabled for incorporating the PID parameters. An algorithm was also developed for proper actuation of IPMC. A laser displacement sensor was used for finding the tip displacement of IPMC membrane. This sensor was activated at 12 V DC and placed in front of fabricated IPMC. This was interfaced with NI-PXI system through an RS485 to the RS-232 converter. The laser displacement sensor continuously checks the displacement position and provides information regarding reaching the desired displacement point. This experimental deflection data was acquired into the NI-PXI through comport and NI visa interface software module in a lab-view VI. Simultaneously, digital analog card (DAQ) assistant of NI-PXI system confirms the actuating voltage from the programmable power supply. The bending responses of SPEES-SWNT-Pt membrane actuator are shown in Fig. 14. The maximum deflection of SPEES-SWNT-Pt IPMC was shown up to 16 mm at 4.5 V DC. The multiple experiments were conducted to investigate the hysteresis behavior, and the average value of deflections at different voltage was plotted as shown in Fig. 15. To check the performance of SPEES-SWNT-Pt IPMC membrane, we have conducted experiments 4 × 10 (40) times as given in supplementary data (Supplementary Tables 2 to 4). The deflection values for the first trial are provided in Table 3. The average value of each 10 experiments was taken as one trial to observe the hysteresis behaviors at multiple repeat (40 times). Moreover, four trials were plotted as shown in Fig. 15. After multiple experiments for deflection behavior by applying voltage, it was found that IPMC did not provide the same behavior which may be due the partial evaporation of inner solvent by electrolysis under applied voltage or natural evaporation. This may be the main reason behind the hysteresis occurred in the fabricated SPEES-SWNT-Pt-based IPMC actuator. To reduce this hysteresis (error), a proportional integral derivative (PID) control system was incorporated in the control system. By enabling this features and setting up the PID gain parameters, the hysteresis was reduced. For this purpose, an algorithm was also developed, where the controller bandwidth was set by tuning the frequency. When the frequency in the controller was increased, the time duration of actuation of SPEES-SWNT-Pt-based IPMC decreases but at the same time the stability of the IPMC was also decreases. In order to achieve the fast response of IPMC, by proper tuning of gains in the PID controller the frequency was set. This reveals the steady position between the response time and stability. In this way, the hysteresis was reduced upto 80%.

**Table 3:** Deflection data of SPEES-SWNT-Pt IPMC membrane at different applied voltages.

Deflection (mm)	Voltage									
	0 V	0.5 V	1.0 V	1.5 V	2.0 V	2.5 V	3.0 V	3.5 V	4.0 V	4.5 V
d1	0	0.6	2.5	6.0	8.0	10.5	12.6	14.9	15.5	16.0
d2	0	0.7	2.6	6.0	8.2	10.2	12.4	14.2	16.0	16.3
d3	0	0.5	1.9	5.8	8.5	10.6	12.6	14.0	15.4	16.2
d4	0	0.8	2.0	5.5	7.8	10.1	12.7	13.8	15.7	16.1
d5	0	0.7	2.1	5.8	7.6	10.9	12.1	14.5	15.8	15.8
d6	0	0.9	1.8	5.4	7.4	10.0	12.9	14.3	15.4	15.9
d7	0	0.4	1.9	5.8	7.6	10.3	12.4	14.7	15.9	16.0
d8	0	0.6	1.8	5.8	7.5	10.0	12.2	14.6	15.2	16.1
d9	0	0.7	2.2	5.5	7.3	10.2	12.0	14.0	15.4	16.2
d10	0	0.5	2.1	5.8	7.8	10.0	12.0	13.9	15.8	16.0

**Figure 16:**
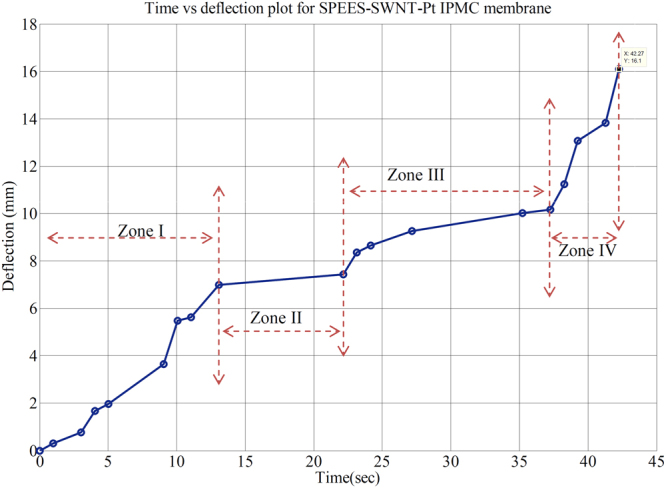
Bending response of SPEES-SWNT-Pt IPMC membrane actuator with time at 4.5 V DC.

The bending response of SPEES-SWNT-Pt-based IPMC overtime at 4.5 V DC is shown in Fig. 16. The time vs. deflection analysis reveals that bending behaviour of fabricated IPMC membrane increases with time and the maximum tip displacement after 42 sec was found to be 16 mm at 4.5 V. As clearly shown in Fig. 16, at the first zone (Zone I), the deflection of SPEES-SWNT-Pt IPMC membrane increases with steady-state continuous upto 13 sec which may be due to high PC and a current density of IPMC at this stage. After that, at the second zone (Zone II) the deflection was constant from 13 to 23 sec. But due to high storage capacity, high PC and IEC of the composite polymer based IPMC the deflection again increases in the third and fourth zone up to 16 mm after applying 4.5 V DC for 42 sec.

**Figure 17:**
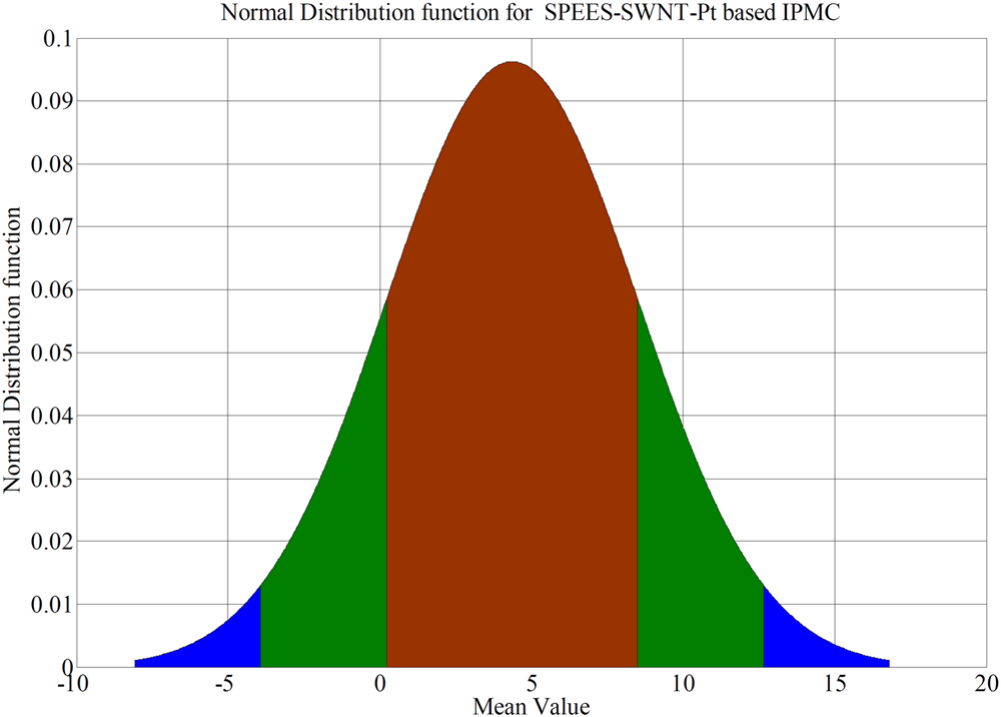
Normal distribution curve for SPEES-SWNT-Pt-based IPMC membrane actuator.

A digital weighing/load cell (Model: Citizen CX-220, Make: India) was taken for load characterization of IPMC membrane, where the tip of SPEES-SWNT-Pt IPMC touches the pan of load cell during applied voltage through NI-PXI system (Supplementary Fig. 3).The maximum load carrying capability for SPEES-SWNT-Pt IPMC based IPMC membrane was upto 1.03 g at 4.5 VDC. In order to find the repeatability, the several trials were conducted and the force data were noted for ten times as given in Table 4. The mean force value was calculated and the normal distribution was found as given in Table 4. The normal distribution for the SPEES-SWNT-Pt IPMC was plotted as shown in Fig. 17. The narrow shape of normal distribution suggested that the fabricated IPMC have less error and great repeatability of the forced behaviour at 4.5 V DC.

**Table 4:** Experimental force data of SPEES-SWNT-Pt IPMC membrane at different voltages.

Force (mN)	Voltage								
	0 V	1 V	1.5 V	2.0 V	2.5 V	3.0 V	3.5 V	4.0 V	4.5 V
F1	0	0.1185	0.4086	0.8496	2.8263	4.4923	7.3960	9.3903	10.14202
F2	0	0.1029	0.3204	0.9290	2.5950	3.9082	6.4160	9.8205	10.33116
F3	0	0.2097	0.4076	0.6585	2.6185	4.6618	8.3731	8.5495	10.31548
F4	0	0.1960	0.3077	0.8898	1.9482	4.7598	7.4921	9.2365	10.13712
F5	0	0.1607	0.2361	0.4557	2.4186	3.5711	6.3455	9.8039	10.31058
F6	0	0.1313	0.2293	0.5929	2.9449	3.9053	7.3950	10.0401	10.01756
F7	0	0.1577	0.2077	0.5399	2.3823	4.5785	7.6332	9.8196	10.40858
F8	0	0.1401	0.2381	0.8791	2.3490	4.2796	6.7169	9.6647	10.03128
F9	0	0.1313	0.1960	0.7105	2.3863	4.9450	7.5303	9.2757	10.42818
F10	0	0.1411	0.2067	0.8251	2.4225	3.7867	6.7561	9.5687	10.21258
Operating voltage									4.5 V
Mean									10.2334 mN
Standard deviation									0.0648
Repeatability									90.24%

**Figure 18:**
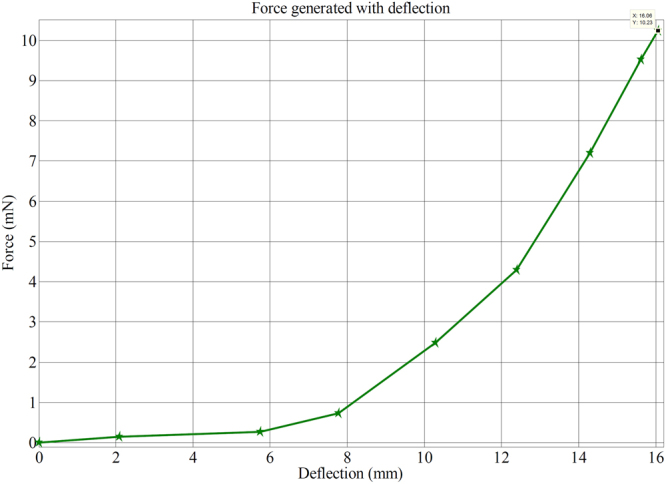
Deflection vs. force behaviour of SPEES-SWNT-Pt IPMC membrane at 0–4.5 VDC.

**Figure 19:**
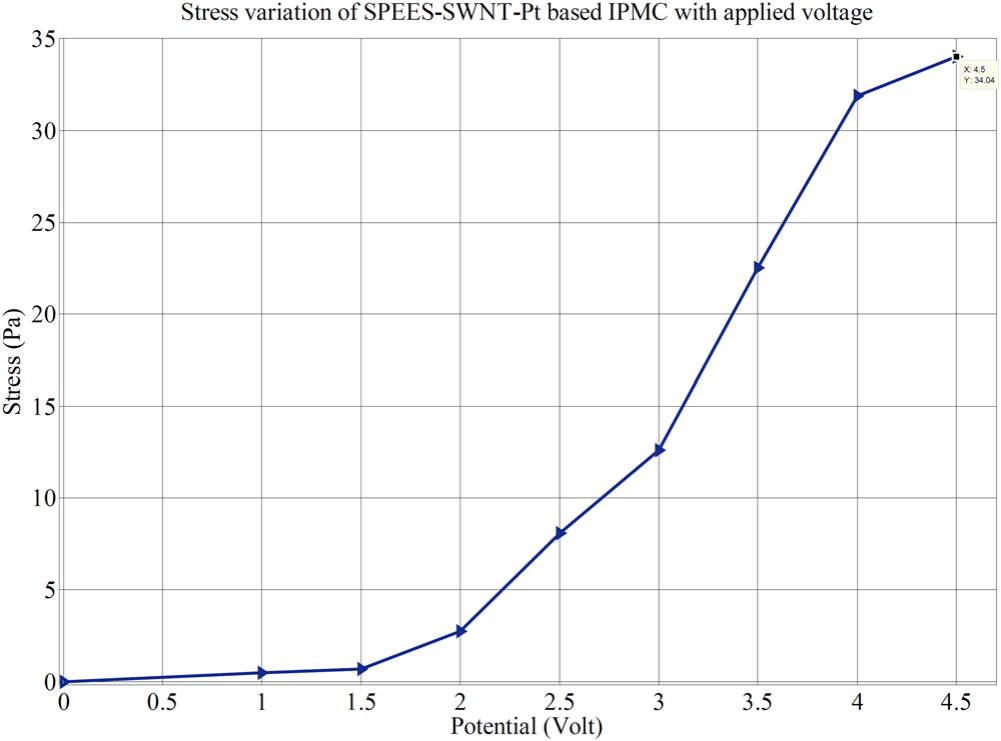
Stress behaviour of SPEES-SWNT-Pt-based IPMC under applied voltages.

The load characterization analysis also confirmed that the bending force was indeed proportional to the electric field like the deflection, where the bending force was found to be increasing with the increase of applied electric field intensity. Furthermore, Fig. 18 clearly shows that the deflection vs. force behaviour of the fabricated IPMC membrane was proportional to the applied electric voltage (0–4.5 V). The electromechanical properties of SPEES-SWNT-Pt-based IPMC membrane was demonstrated by measuring the stress behaviour with respect to the applied potential (Fig. 19). It was found that the maximum stress generated in SPEES-SWNT-Pt composite membrane actuator was 34.04 Pa under an applied voltage (0–4.5 VDC). The comparison of different properties of SPEES-SWNT-Pt-based IPMC membrane actuator with the other reported IPMC actuators is given in Table 5.

**Table 5:** Comparison table of SPEES-SWNT-Pt-based IPMC membrane actuator.

Parameter	SPEES-SWNT-Pt	Sulfonated polyetherimide^56^	Sulfonated polyvinyl alcohol/Py^12^	CNT based actuator^57^	Nafion^58^	Kraton based IPMC^35^
IEC (meq g^−1^)	2.60	0.55	1.20	0.71	0.98	2.00
PC (S cm^−1^)	2.32 × 10^−2^	1.40 × 10^−3^	1.60 × 10^−3^	5.70 × 10^−3^	9.00 × 10^−3^	1.30 × 10^−3^
WU (%)	80.0	26.4	82.3	25.1	16.7	233.0
Current density (A cm^−2^)	4.1 × 10^−3^	5.0 × 10^−4^	5.5 × 10^−3^	—	3.0 × 10^−3^	2.5 × 10^−3^
Tip displacement (mm)	16.0	2.7	18.5	20.0	12.0	17.0

## Conclusions

The present study demonstrated a novel high-performance durable IPMC membrane, based on SPEES, functionalized SWNT, IL composite with Pt electrode, leading to better actuation performances, electrochemical and mechanical properties. The fabricated IPMC membrane exhibited an increased IEC and proton conductivity, which facilitated ion transport within the membrane actuator and therefore resulted in fast response and large deformation. The notable combination of advantages including good electrochemical activity, large bending deformation, high capacitance and flexibility in SPEES-SWNT-Pt IPMC membrane may come from the combining properties of hydrophilic SPEES containing more sulfonic acid groups and functionalization of carbon nanotubes. Moreover, the cost-effective SPEES polymer used was free of fluorine unlike Nafion, thus environmental-friendly and ecologically more acceptable material. Therefore, the proposed actuator can be a promising candidate for wearable electronics, bio-mimetic robots that require eco-friendly and high-performance electroactive soft actuators.

## Electronic supplementary material

Supplementary Information
