# Supplementary material for: Development, Characterization and Electromechanical Actuation Behavior of Ionic Polymer Metal Composite Actuator based on Sulfonated Poly(1,4-phenylene ether-ether-sulfone)/Carbon Nanotubes
Source: Sci Rep. 2018 Jul 2;8:9909. doi: 10.1038/s41598-018-28399-6 (PMC6028589; doi:10.1038/s41598-018-28399-6)
Supplement: Supplementary file 1 — Supplementary Information [file 41598_2018_28399_MOESM1_ESM.pdf]

Development, Characterization and Electromechanical Actuation Behavior of Ionic Polymer Metal Composite Actuator based on Sulfonated Poly(1,4-phenylene ether-ether-sulfone)/Carbon Nanotubes

Ajahar Khan[1], Ravi Kant Jain[1*], Priyabrata Banerjee[2,3], Bhaskar Ghosh[1], Inamuddin[4,5,6] and Abdullah M. Asiri[4,5]

[1]Department of Surface & Field Robotics Group, CSIR- Central Mechanical Engineering Research Institue (CMERI), Durgapur-713209, India
[2]Surface Engineering & Tribology Group, CSIR-Central Mechanical Engineering Research Institute, Durgapur-713209, India
[3]Academy of Scientific and Innovative Research (AcSIR), CSIR-Central Mechanical Engineering Research Institute (CMERI) campus, Durgapur 713209, India
[4]Chemistry Department, Faculty of Science, King Abdul Aziz University, Jeddah 21589, Saudi Arabia
[5]Centre of Excellence for Advanced Materials Research, King AbdulAziz University, Jeddah 21589, Saudi Arabia
[6]Advanced Functional Materials Laboratory, Department of Applied Chemistry, Faculty of Engineering and Technology, Aligarh Muslim University, Aligarh 202002, India
Email: arkhan.029@gmail.com, rkjain@cmeri.res.in

Supplementary Information

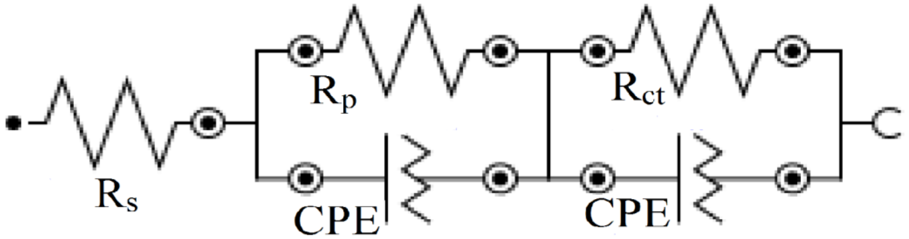

Supplementary Figure 1. Equivalent circuit model to estimate the electrochemical parameters of EIS for SPEES-SWNT-Pt IPMC.

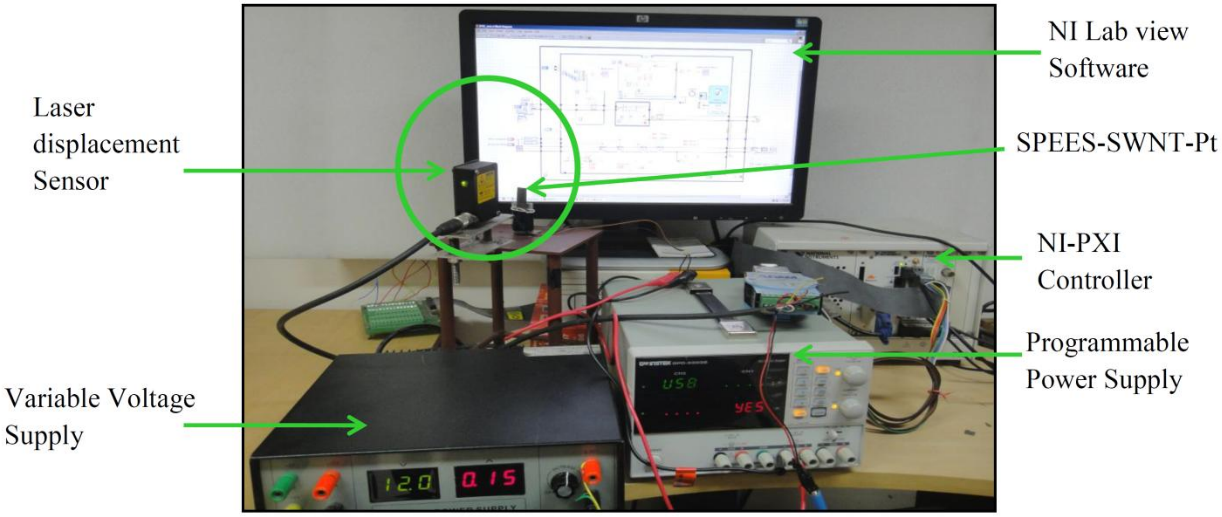

Supplementary Figure 2. Actual testing setup to measure the tip displacement of SPEES-SWNT-Pt based IPMC membranes.

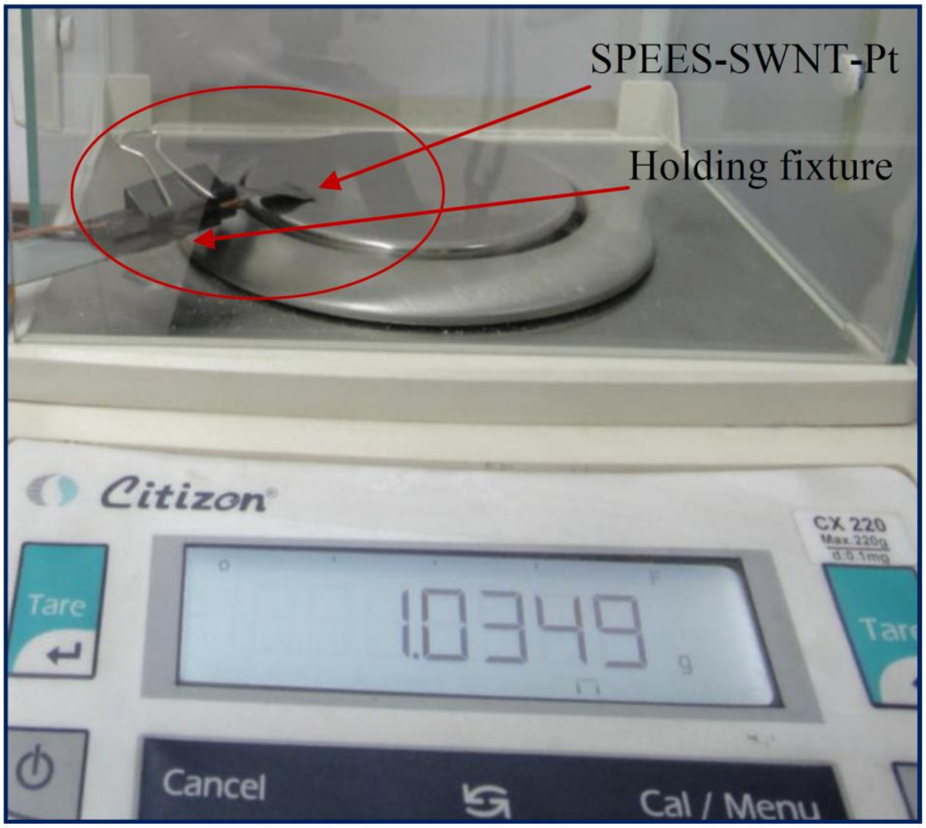

Supplementary Figure 3. Testing setup for load chahracterization of SPEES-SWNT-Pt based IPMC membranes.

Supplementary Table 1. The WU %, IEC and proton conductivity of the SPEES-SWNT-Pt based IPMC membrane.

**Sample**	**WU% At R.T**	**IEC (meq/g)**	**PC (σ) (S/cm)**
SPEES-SWNT-Pt	80 (24 h)	2.5	2.321×10^{-2}

Supplementary Table 2. Experimental tip displacement data of SPEES-SWNT-Pt IPMC membrane for trial 2.

Deflection (mm)	Voltage (V)									
	**0 V**	**0.5V**	**1.0V**	**1.5V**	**2.0V**	**2.5V**	**3.0V**	**3.5V**	**4.0V**	**4.5V**
d1	0.4254	1.3229	6.0443	9.4427	8.5338	11.6542	13.7217	16.5909	15.6132	16.0373
d2	0.4585	1.0145	3.9387	9.1628	9.154	10.9343	13.084	16.8455	16.0365	16.3267
d3	0.4176	0.5401	5.0938	7.9603	9.0125	14.4567	15.3167	16.5415	15.4406	16.2137
d4	0.2084	1.386	5.229	8.4758	9.9431	13.1343	16.3208	15.907	15.7459	16.1159
d5	0.1131	1.1691	2.3016	8.768	7.8275	14.7131	13.5123	15.2675	15.8031	15.9121
d6	0.5457	1.2788	3.415	7.1846	9.2801	12.6933	13.3019	16.3539	15.4751	15.9583
d7	0.0296	0.8226	5.7726	9.2121	11.5428	11.0465	15.5818	17.7025	15.967	16.0886
d8	0.5968	0.8302	3.4626	7.7842	10.2373	11.1629	14.0795	16.2845	15.2433	16.1368
d9	0.3026	1.2562	3.5262	9.3043	10.7943	10.4335	14.7578	16.959	15.4499	16.2009
d10	0.6179	0.7506	4.6533	9.001	9.7909	10.1768	14.7407	16.6543	15.8548	16.0729

Supplementary Table 3. Experimental tip displacement data of SPEES-SWNT-Pt IPMC membrane for trial 3.

Deflection (mm)	Voltage (V)									
	**0 V**	**0.5V**	**1.0V**	**1.5V**	**2.0V**	**2.5V**	**3.0V**	**3.5V**	**4.0V**	**4.5V**
d1	0.6385	1.2247	6.0874	8.9696	10.4362	10.5598	14.9469	16.1733	15.5022	16.0426
d2	0.5284	0.9746	5.9491	6.2222	10.9232	10.374	13.0454	15.3304	16.0975	16.3765
d3	0.5332	0.9236	5.0159	8.8435	12.4699	11.3246	14.7257	17.2966	15.5252	16.2476
d4	0.4106	1.3548	5.168	7.3631	11.7925	10.9058	14.5598	15.1751	15.7616	16.1338
d5	0.1461	0.8566	3.6018	5.9809	10.645	13.7915	13.6256	18.0051	15.89	15.8777
d6	0.3813	1.3979	2.4122	8.7846	8.5056	11.7802	13.2747	15.3774	15.4835	15.9154
d7	0.4327	0.4001	3.316	6.4621	11.4348	13.6986	13.4417	17.8169	15.9324	16.0196
d8	0.6406	0.9969	4.393	6.2626	9.1524	11.5668	13.5499	17.0488	15.3119	16.1135
d9	0.1569	1.1407	5.946	6.5916	8.1707	13.5666	13.5078	14.0722	15.441	16.2443
d10	0.4081	0.5054	2.4751	7.0637	10.3289	13.0084	12.5879	16.7198	15.9318	16.1159

Supplementary Table 4. Experimental tip displacement data of SPEES-SWNT-Pt IPMC membrane for trial 4.

**Deflection (mm)**	**Voltage (V)**									
	**0 V**	**0.5V**	**1.0V**	**1.5V**	**2.0V**	**2.5V**	**3.0V**	**3.5V**	**4.0V**	**4.5V**
d1	0.2699	1.3382	6.3596	8.5575	9.3792	11.5994	16.4615	15.0399	15.6112	16.1303
d2	0.1008	0.8031	5.1899	9.9883	8.4737	10.4688	15.4127	17.7693	16.0323	16.4046
d3	0.4998	0.7006	3.9458	6.4422	11.1519	11.9533	16.1999	16.1196	15.4576	16.2464
d4	0.5855	1.0967	3.4533	8.0025	8.7913	10.3025	15.1304	17.0799	15.8096	16.1664
d5	0.6316	1.4795	5.2184	7.0528	10.6757	11.3111	14.8986	16.3163	15.8202	15.8338
d6	0.211	1.1674	4.983	5.7453	8.6134	10.2917	14.4872	15.7063	15.5003	15.9675
d7	0.3404	1.1056	2.0174	9.3582	9.2996	13.9706	13.3061	17.7726	16.016	16.0104
d8	0.3152	0.7863	2.0128	8.1971	7.7266	11.2653	12.7327	16.0618	15.2664	16.1074
d9	0.477	0.8726	3.5125	6.7301	7.4252	12.6014	12.6999	16.2587	15.4755	16.284
d10	0.604	1.2774	5.3452	6.482	8.5572	11.5324	15.8089	17.5065	15.9127	16.0816
